# Transient response of magnetorheological fluid on rapid change of magnetic field in shear mode

**DOI:** 10.1038/s41598-022-14718-5

**Published:** 2022-06-23

**Authors:** Michal Kubík, Josef Válek, Jiří Žáček, Filip Jeniš, Dmitry Borin, Zbyněk Strecker, Ivan Mazůrek

**Affiliations:** 1 Faculty of Mechanical Engineering, Brno University of Technology, Brno, Czech Republic; 2 Chair of Magnetofluiddynamics, Institute of Mechatronic Engineering, Technische Universitat Dresden, Dresden, Germany

**Keywords:** Electrical and electronic engineering, Actuators, Fluidics, Nanoscale materials, Characterization and analytical techniques

## Abstract

The transient behaviour of magnetorheological (MR) devices is an important parameter for modern semi-actively controlled suspension systems. A significant part of the MR device response time is the MR fluid response time itself. A significant factor is the so-called rheological response time. The rheological response time is connected with the structuring particle's time and the development of shear stress in MR fluid during the deformation. The main aim of this paper is to experimentally determine the rheological response time of MR fluid and evaluated the effect of shear rate, magnetic field level, and carrier fluid viscosity. The unique design of the rheometer, which allows the rapid change of a magnetic field, is presented. The rheological response time of MRF 132-DG and MRC-C1L is in the range of 0.8–1.4 ms, depending on the shear rate. The higher the shear rate, the shorter the response time. It can be stated that the higher the magnetization of the MR fluid, the lower the response time. The higher the viscosity, the higher the rheological response time. The measured data of rheological response time was generalized and one master curve was determined.

## Introduction

Magnetorheological (MR) fluid is the suspension of fine, non-colloidal, low-coercivity, high-magnetizable particles in a carrier fluid. These particles are usually made of carbonyl iron and have a spherical shape due to their durability and tribological properties. The continuous phase of MR fluids is typically silicon or synthetic hydrocarbon oils^1^. The lowest possible viscosity of the continuous phase is required, but this significantly affects the sedimentation stability^2^. MR fluid also contains several additives that affect rheological^3^, tribological^4^, or sedimentation stability^5^. When the MR fluid is energized by the magnetic field, the ferromagnetic particles are magnetized and form chain-like structures in the direction of the magnetic field^6^. The rheology of MR fluid in activated state is characterized by pre-yield and post-yield regime. In the pre-yield regime, the MR fluid exhibits viscoelastic behaviour. The complex modulus *G* is a magnetic field *H* and particle concentration dependent. The shear stress *τ* in the fluid can be described by the equation below

$$\tau = G\gamma ,\,\tau < \tau_{0} (H)\,{\text{and}}\,\dot{\gamma } = 0$$

where $\gamma$ is shear strain, $\dot{\gamma }$ is shear rate and *τ*_*0*_(*H*) is MR fluid yield stress. The post-yield regime is usually described by Bingham model as follows:

$$\tau (H) = \tau_{0} (H) + \eta \dot{\gamma }$$

where *τ*(*H*) is shear stress, $\eta$ is Bingham viscosity, and *H* magnetic flux intensity. It is the simplest model that can described this behaviour. The MR dampers^7,8^, clutches/brakes^9^, or seals^10,11^ take advantage of the unique behavior of MR fluid.

The transient behaviour (transient response) of MR fluid is an important parameter for modern magnetorheological devices working with real-time control^12,13^. The MR fluid response time is composed of other partial response times which are differently important depending on the operating conditions and the method of MR fluid loading. The response time of MR fluid can be divided into (1) hydrodynamic response time, (2) particle structure development response time, and (3) rheological response time.

The research studies of Sherman^14^ or Goldasz et al.^15^ show that MR valve pressure drop due to MR fluid yield stress decreases with the increasing gap velocity. At high velocities, this pressure drop is approaching to be zero. This statement is based on CFD (computational fluid dynamics) simulations. This phenomenon is related to transient rheology connected with the development of the velocity profile in the gap and is often referred to as the hydrodynamic fluid response time. Goncalves et al.^16^ experimentally determined that the hydrodynamic response time is 0.73 ms for magnetic field 100 kA/m and 0.53 ms for magnetic field 200 kA/m. The commercial MRF-132LD (Lord Corp., USA) was used in this study. Kubík et al.^17^ published similar study. This team measured the hydrodynamic response time of MR fluid MRF-132DG (Lord Corp., USA) and ranges from 0.4 to 1 ms for a selected gap size and a range of magnetic field stimuli. The velocity profile development mechanism is similar for MR fluid and electrorheological (ER) fluid^18^. However, ER fluid show faster response time than MR valve. ER fluid is the suspension of fine electrically active particles in fluid. This fluid exhibits a rapid increase of fluid yield stress under the application of an electric field. Gavin et al.^19^ modelled the transition from a fully developed Bingham profile to a Newtonian flow for ER fluid. The yield stress of ER fluid was assumed to drop to zero quicker than the dissipation energy due to the development of the velocity profile^19^. It can be stated that this hydrodynamic response time is connected with high shear rates or fast changes of the magnetic field in valve mode.

The particle structure development response time is related to the time needed for the structuring of particles in the direction of the magnetic field without the flow conditions of the MR fluid. Jolly et al.^20^ proposed an experimental method that microstructure formation time can be deduced from the transient changes in the relative magnetic permeability of the MR fluid. The chained particles are assumed to have a higher magnetic permeability than the dispersed. Two-time responses were observed^20^. The first attributes the connection with the transfer of particles into diverse chains (pair formation) and the second (an order of magnitude slower) connection with the migration of these initial chains into longer and stronger structures. The response time was between 5 and 10 ms. A similar measurement method was also published by Horváth et al.^21^. Pei et al.^22^ stated that the response time of dry MR fluid was in the order of µs by the model. This statement is based on simulation results.

The rheological response time is connected with the structuring particle's time and the development of shear stress in MR fluid during the deformation (flow). Sherman et al.^23^ create a chain model of MR fluid. This model is based on one million particles. One result of this paper is the shear stress time history on the step change of a magnetic field. For this data, the rheological response time can be determined as roughly 0.4 ms. The MR fluid had a volume particle fraction of 25% and was under the shear rate of 500 s^−1^. Laun and Gabriel^24^ determined the response time of MR fluid of 2.8 ms. They used sinusoidal excitation and the determined time lag between magnetic flux density and shear stress. Kikuchi et al.^25^ examined the response time to a step electric current and introduce non-dimensional response time parameter. It can be expected that the mechanism of chain formation in Electro-rheological (ER) fluids and MR fluids is similar. Koyanagi et al.^26^ developed a method for a measurement response time of ER fluid. This team experimentally determined the response time as 0.95 ms.

The information about the transient behaviour of MR fluid is limited. This issue is becoming more important due to the development of MR devices with a short response time^7,27^, where the limiting part is now the MR fluid itself. The current design of the MR damper achieved a response time of about 1.2 ms. In the current state of the art, more studies can be found dealing with the response time of MR fluid^7,12^ than is presented above. In these several cases, the authors measured the time constant of measuring devices instead of the time constant of MR fluid^14^. The rheological response time of MR or ER fluid was just experimentally determined in studies^24,26^. Both studies presented response time just for one experimental condition. The main aim of our paper is to experimentally determine the rheological response time of MR fluid and evaluated the effect of shear rate, magnetic field level, and carrier fluid viscosity. Our results will be compared with the published analytical approach^14^.

## Materials and methods

### Description of the measured phenomenon and measuring methods

**Figure 1:**
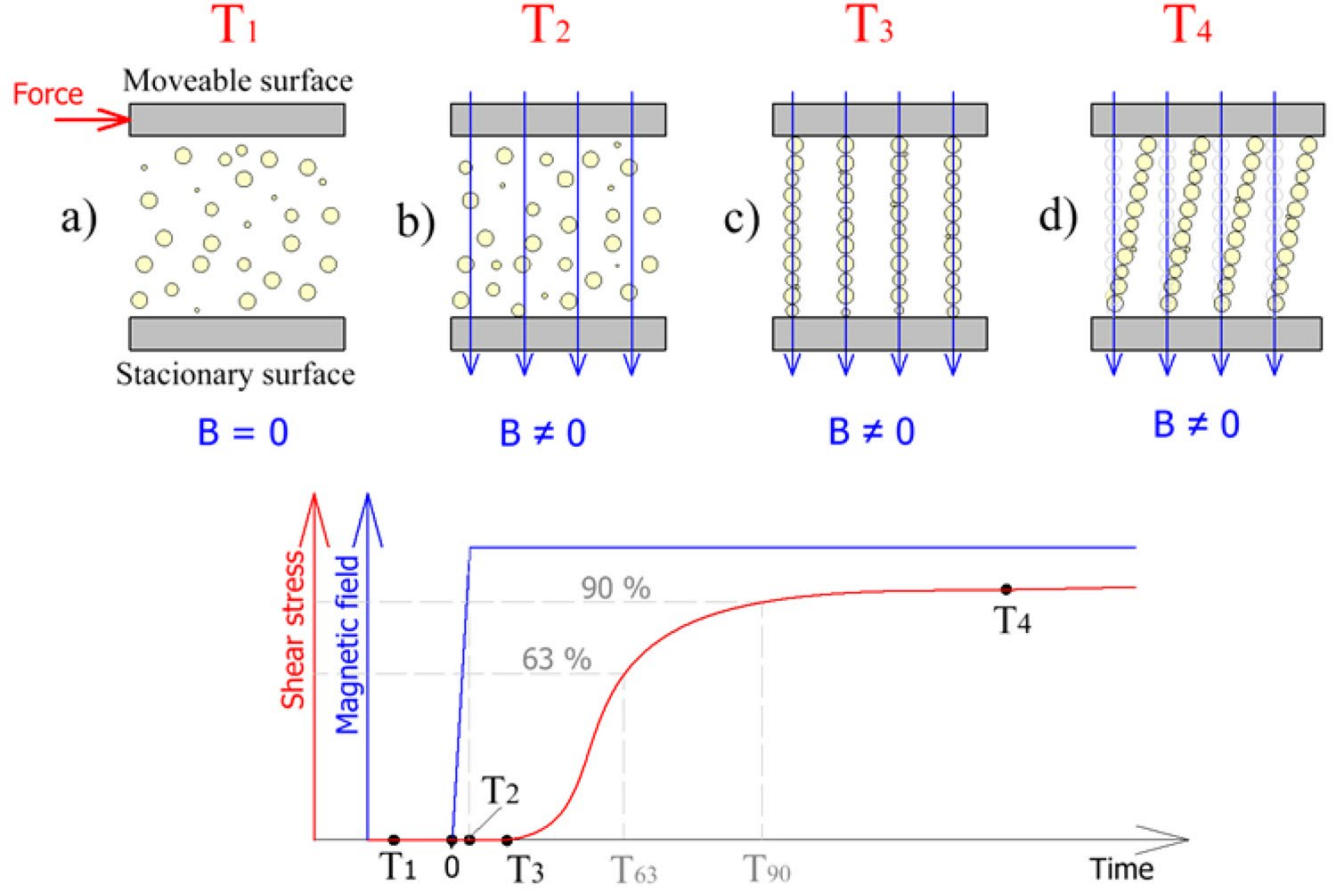
Demonstrating measured method and determination of time constants.

The aim of the measurement is to experimentally determine the time constant of MR fluid in the shear mode (from the increase in shear stress τ) on a rapid change in the magnetic field *B*. The procedure of the experiment is described in Fig. 1. At time T_1_, the MR fluid is loaded by given shear rates and the magnetic field is off. At time 0, the magnetic field is activated and, at time T_2_, the magnetic field is already at the maximum value. However, until time T_3_, the shear stress remains at the same level as at time T_1_. In the author's opinion, this delay is associated with particle structure formation in the MR fluid. In reality, there are no separate single chains. That is just a tentative simplification. At time T_4_, there is a rapid increase in shear stress in the MR fluid due to the deformation of the particle structure. This is shown as tilting chains in the shear direction but the mechanisms of structure fracture are more complex. Generally, the simplest dynamic system, that can serve as an approximation of the transient behavior of MR fluid is a first-order system. The transient response is expressed by the time constant T_63_ (primary response time), which determines the time when monitored torque (calculated shear stress) achieved 63.2% of the final controlled value (steady-state). This approximation can be used for the description of the dynamic behaviour of MR actuators^28^. In the case of rheology measurement, the MR fluid can be described by a simple Maxwell model and by Bingham constitutive equation. For step change on magnetic field, the excepted shear stress response *τ*(*t*) would be:

1 $$\tau \left( t \right) = \tau_{0} \left[ {1 - e^{{ - \frac{t}{{T_{63} { }}}}} } \right] + \eta \dot{\gamma }$$

where *t* is time. More names for a variable T_63_ can be found in the literature as switching time^24^, response time^17^ or rheological response time^14^. However, the transient response of MR fluid exhibits different behaviour than the first-order system, see Fig. 5. Therefore, we decided to determine those time constants in our paper: (1) first-order time constant T_63_ (0–63.2%) and (2) rise time T_90_ (0–90%), see Fig. 1. This response time were so-called rheological response time because it is connected with changes in the rheology of MR fluid. Those time constants were selected due to a suitable comparison of our experimental data with results from published papers.

### Experimental test rig

**Figure 2:**
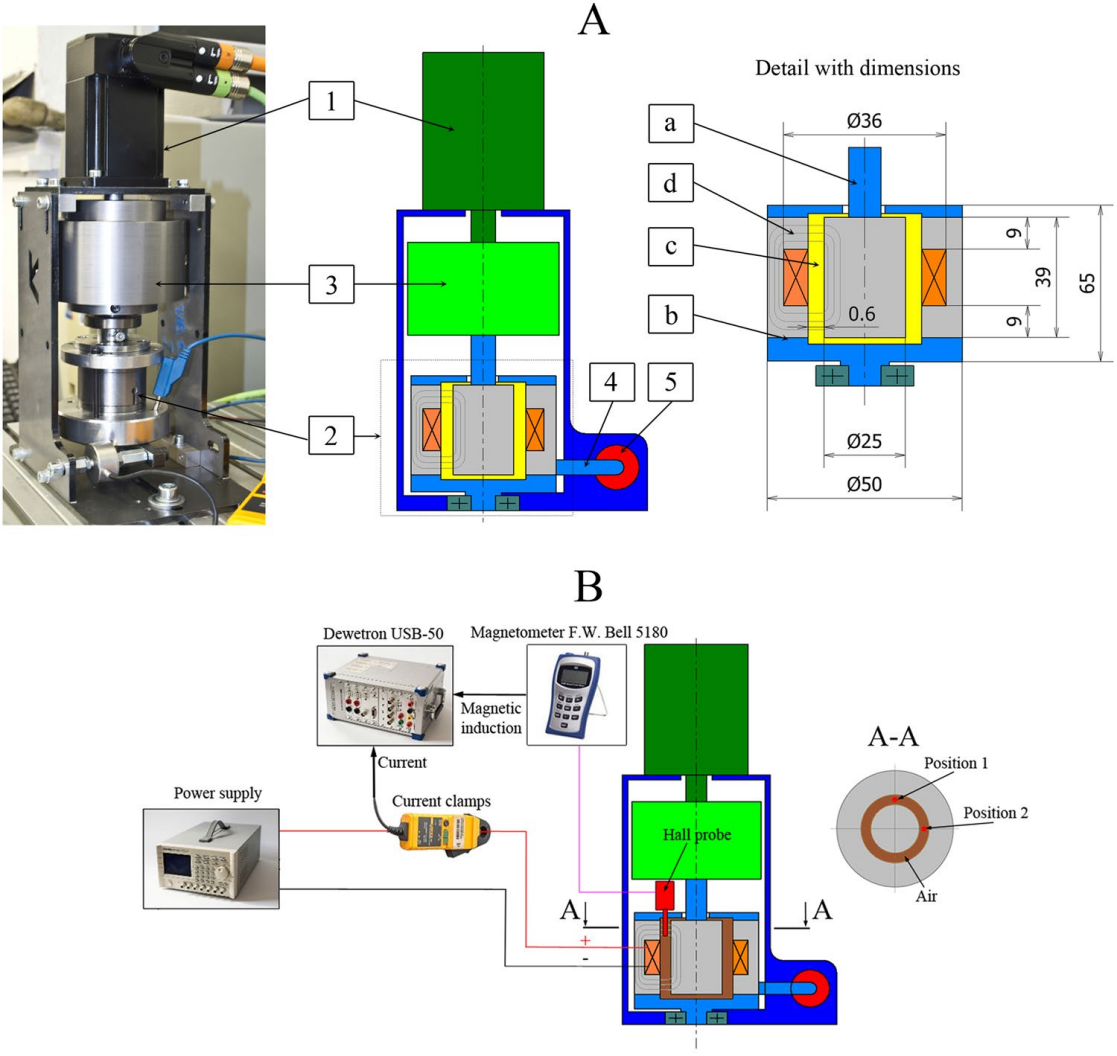
(**A**) Rheometer design with important dimensions (grey, soft magnetic composite material; orange, copper; light blue, aluminium; yellow, MR fluid sample, green, steel) and (**B**) magnetic flux density measurement in the gap.

The experimental test rig is composed of an electric motor with encoder (1), developed rheometer (2), load inertia (3), lever (4), and force sensor (5), see Fig. 2A. The load inertia accumulated energy to stabilize rotation during the activation of MR fluid using the magnetic field (an increase motor load). The load inertia had 4.6 kg (moment inertia 5900 kg/mm^2^). The whole system is mounted rotationally and the torque is measured by a force sensor on the lever (52 mm). The homemade rheometer is composed of the rotor (a), stator (b), and MR fluid sample (c), see Fig. 2. The electromagnetic coil creates the magnetic flux (d) in the magnetic circuit (show in grey). The gap size was 0.6 mm, see Fig. 2. The transient behaviour of the rheometer is fundamental for the precise measurement of MR fluid response time. The response time of hardware (rheometer) has to be as short as possible and two main sources were identified in the literature: (1) eddy currents in the magnetic circuit^7^, and (2) inductance of the rheometer electromagnetic coil^7^. In our rheometer, we used soft magnetic composite (SMC) material (trademark Sintex) for the magnetic circuit to eliminate eddy currents. SMC material is magnetic conductive and electric non-conductive (resistivity 280 µΩm). The suitable design of a magnetic circuit with our patented current controller allows a rapid increase of electric current on the electromagnetic coil (*T*_63*I*_ = 0.21 ms).

### Methodology measurement

The aim of the experiments was to determine shear stress in MR fluid and magnetic field over time. The shear stress *τ* was calculated from area and torque which was measured indirectly based on data from the force sensor (MEG20) on the lever, see Fig. 2. The force sensor measuring range was 0–200 N. The force range (deformation) was chosen to maximize system rigidity and only the first 10% of the range was used for measurement. The magnetic field in the gap corresponds with the electric current course and was measured by Fluke i30 current clamps. These two signals were recorded and conditioned with a sampling frequency of 200 kHz by the Dewetron USB-50 analyzer. The MFG-2120MA signal generator generates a square wave voltage signal which inputs to the current controller at a frequency of 1 Hz. Our developed current controller generates an electric current on the electromagnetic coil with over-voltage up to 100 V. The measurement procedure was as follows: (1) 10 s measurement without magnetic field, and (2) 10 s measurement with the application of the magnetic field. This procedure was necessary for the elimination of non-constant friction forces in the rheometer and viscous forces. Those phenomena can significantly complicate the subsequent evaluation of response time. The experiments were conducted 5 times under the same conditions. The data was not filtered but averaged from raw data. Then, the ramp data was normalized. All measurements were performed at 25 °C ± 1 °C.

### Methodology evaluation of response time

The measured response time of the magnetic field (electric current) achieved a value of τ_63I_ = 0.21 ms and τ_90I_ = 0.33 ms, see Fig. 4. In several cases of the transient behaviour of MR actuators, this time can be expected as a step change. In our case, we cannot make this simplification because the expected response time of MR fluid from published models^14^ is in the same time scale (roughly 1.5 ms). Therefore, it was necessary to determine the transfer function between the measured magnetic field and shear stress in MR fluid. We used a process model for describing the MR fluid transient response. The process model is popular for describing system dynamics in many industrial applications^29^. We used the so-called simple SISO (Single Input, Single Output) process model which is described by this transfer function:

2 $$sys = \frac{{K_{p} }}{{1 + T_{p} s}}e^{{ - T_{d} s}}$$

where $K_{p}$ is the proportional gain, $T_{p}$ is the time constant, and $T_{d}$ is dead time. A similar approach was used in study^26^. The Matlab System identification toolbox was used for the identification of constants. The length of the evaluated section was 20 ms.

### Magnetorheological fluid samples

The commercial MR fluid MRF-132DG supplied by Lord Corp., MR fluid MRHCCS4-A and MRHCCS4-B supplied by Liquids Research, and MRC-C1L supplied by CK Materials were chosen as the samples, see Table 1. These fluids were chosen because they have a similar particle size and a different viscosity of the carrier fluid.

**Table 1:** MR fluid samples.

	MRF-132DG	MRHCCS4-A	MRHCCS4-B	MRC-C1L
Solid content by weight (%)	80.98	70	80	80
MR fluid viscosity at 40 °C (Pa s)	0.114	0.167	0.237	0.108
Carrier fluid viscosity at 40 °C/25 °C (Pa s)	0.011/0.018	0.03/0.051	0.03/0.051	0.008/0.011
Average particle size (µm)	Spherical 2.1	Spherical 1.8	Spherical 1.8	Spherical 1–5

The viscosity listed in the table was measured by the Haake Rotovisco 1 rheometer, and determined as a slope between 400 and 800 s^−1^. It should be noted that carrier fluid of MR fluids exhibits Newtonian behaviour but MR fluids are in general non-Newtonian. The particle sizes were measured by a scanning electron microscope, FEG SEM ZEISS Ultra Plus, and analysed by script using tools for picture analysis in Matlab. However, the information about particle size of MRC-C1L was taken from study^30^.

### Magnetic model and experimental validation settings

The data from the magnetic model are necessary for the generalization of measured response time data. The magnetic model was created in Ansys Electronics Desktop 19.2. The geometry of the magnetic circuit was simplified. The magnetization curve of the magnetic circuit material (SMC material) was extracted from the datasheet of the supplier. The electromagnetic coil (70 turns) carrier was made of plastic with relative permeability 1. The lids were made of aluminium also with a relative permeability of 1. The magnetization curve of MR fluid was taken from a MR fluid supplier datasheet. This model was necessary for the calculation of MR fluid magnetization *M,* which is an important input for the calculation of Mason number M_n_. The Mason number M_n_ is the ratio of magnetic forces to viscous forces and is usually used for the description of MR fluid's behaviour at the microscopic level^31^. The magnetometer F.W. Bell 5180 with an ultrathin transverse probe (STB1X-0201) was used for magnetic measurement. The Fluke i30 current clamps were used for electric current measurement. These two signals are recorded and conditioned with a sampling frequency of 100 Hz by a front-end Dewetron USB-50-USB2-8 connected to the laptop, see Fig. 2B.

## Results and discussion

### Magnetic model validation

**Figure 3:**
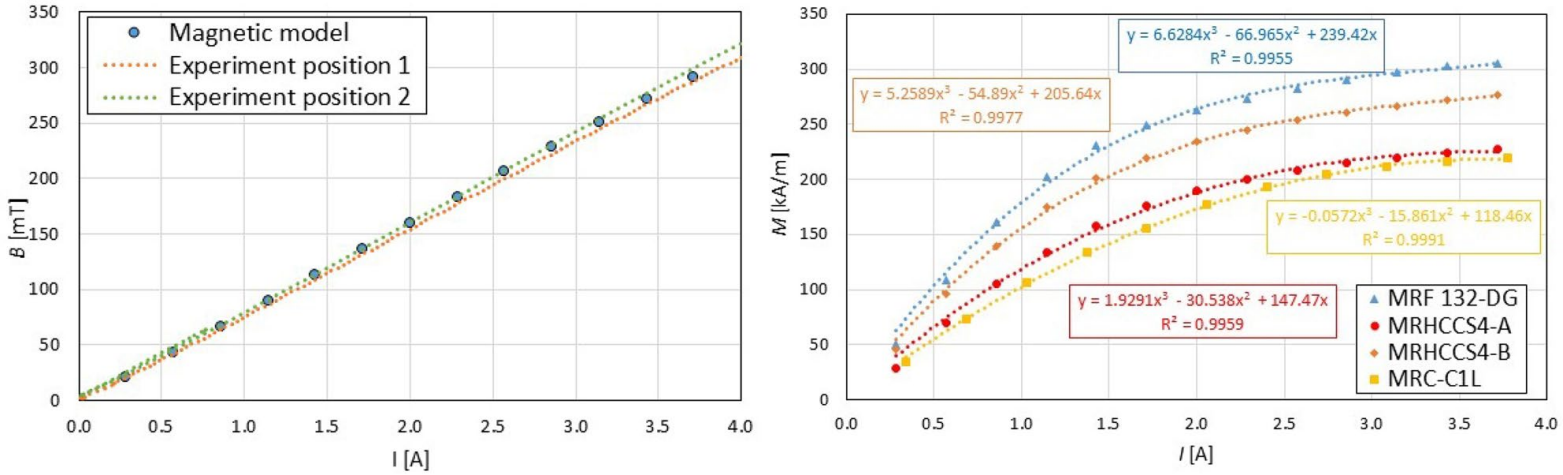
The results from the magnetic model and experiment for air gap (left), The calculated magnetization M dependency of electric current I on the coil for different MR fluids (right).

The comparison of results of magnetic flux density *B* over the electric current from the magnetic model and experiment with air in the gap can be seen in Fig. 3, left. Magnetic flux density measurements in the gap were performed for two positions that are perpendicular, see Fig. 2B. The agreement between model and experiment is acceptable. This experimentally verified model was used for the calculation of magnetization *M* in the gap with MR fluid. The results can be seen in Fig. 3 right. This data is necessary for the calculation of Mason number M_n_.

### Control electric current signal

**Figure 4:**
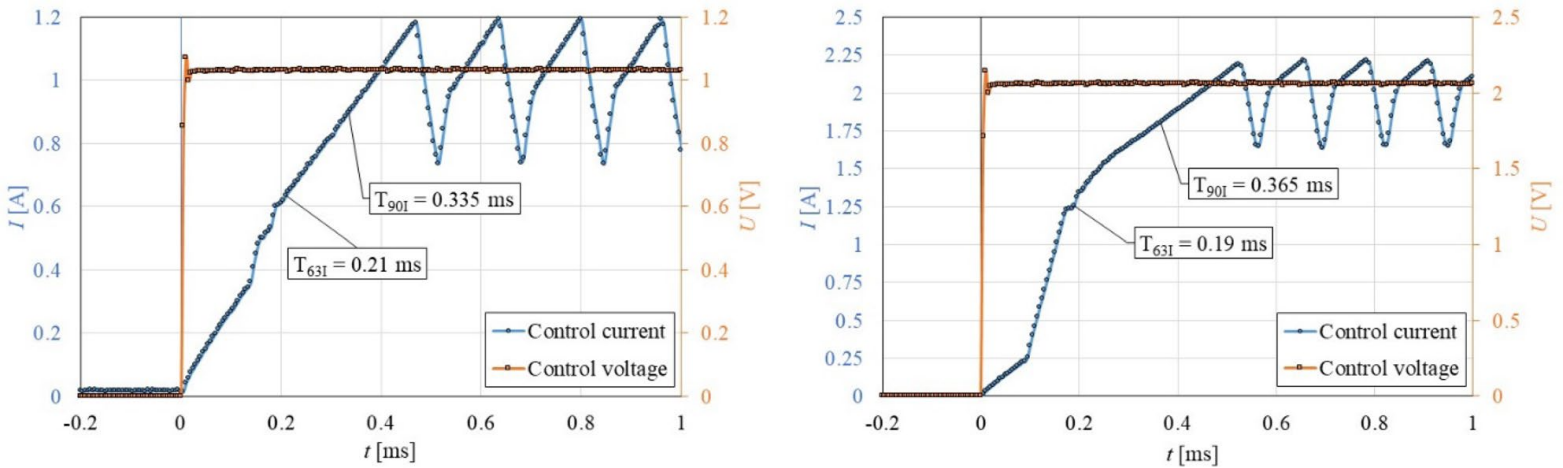
The course of the electric current I in the time t for the final value of electric current 1 A (left) and 2 A (right).

First of all, it was necessary to precisely describe the excitation of MR fluid. It can be assumed that the course of magnetic flux density in the MR fluid copies the course of an electric current due to the elimination of eddy current in the magnetic circuit. This is ensured by a special design of the rheometer. The course of the electric current *I* in time *t* can be seen in Fig. 4 for two levels of electric current *I*. A fast rise in the electric current *I* is achieved by connecting a higher voltage than results from the Ohm law (over-voltage method). When the required electric current value is achieved, the current controller starts to regulate at a frequency of 8 kHz. Therefore, the electric current exhibits oscillations in time *t* after 0.5 ms. Next, reducing the electric current response time was not possible due to the available current controller (maximum 100 V) and rheometer design (coil inductance). The response time (90%) of the electric current achieved a value of $T_{90I} = 0.335$ ms for electric current 1 A and a value of $T_{90I} = 0.365$ ms for electric current 2 A, see Fig. 4.

### The selected courses of MR fluid shear stress in time

**Figure 5:**
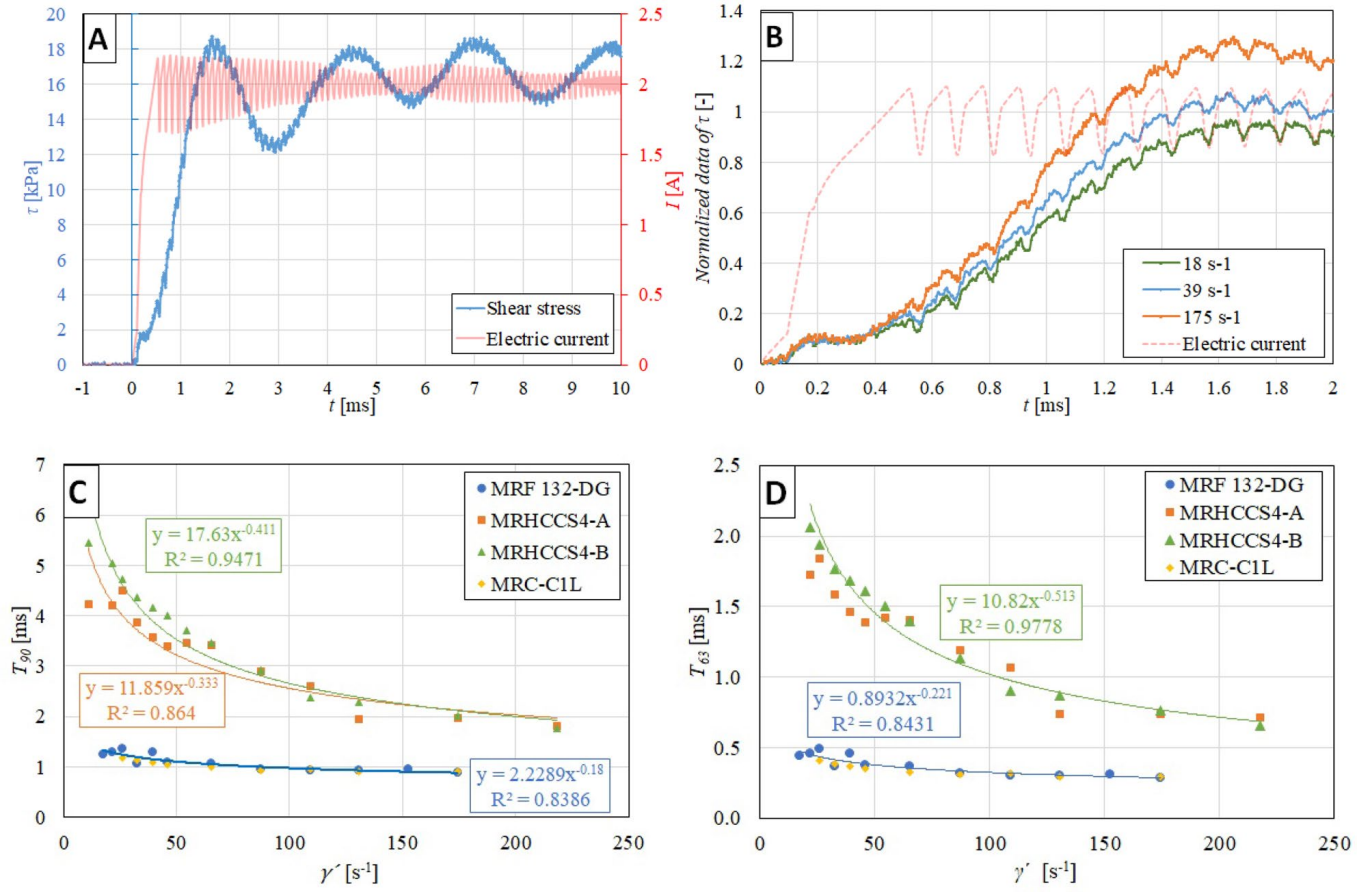
(**A**) The selected shear stress and electric current course over time for shear rate 39 s^−1^ and MRF 132-DG, (**B**) the effect of shear rate on the normalized course of shear stress in MRF 132-DG; The effect of shear rate on response time 90% (**C**), 63% (**D**) for different MR fluids at the same electric current excitation of 2 A.

The Fig. 5A show the course of shear stress $\tau$ and electric current *I* over time *t*. The course of shear stress $\tau$ exhibits oscillations with a constant frequency of 360 Hz, which is connected with the natural frequency of some part of the rheometer. This hypothesis was verified by measurements using an accelerometer and evaluation based on FFT (Fast Fourier transform). The measured frequency was 337 Hz ± 4.88 Hz. The right of Fig. 5B the effect of shear rate $\dot{\gamma }$ on the course of shear stress *τ*. With the increase of shear rates $\dot{\gamma }$, the response time decreases. An initial dead time of 0.4 ms can also be seen, which is independent of the shear rate level, see Fig. 5B. It should be noted that this phenomenon may be associated with an increase in the magnetic field. The previous study^26^ measured a dead time of 0.5 ms for ER fluids and dead time of 0.6 ms for MR fluid^7^, which is consistent with our experiments. We assume that the measured dead time of 0.4 ms is related to the chaining of ferromagnetic particles (microstructure formation) in the MR fluid.

### The effect of shear rate on the rheological response time

The response times shown in Fig. 5C were determined from the experimental data and evaluated according to a process model. The relationship between response time and shear rate $\dot{\gamma }$ is nonlinear. The measured data of response time can be fitted by a power-law function, see Fig. 5C. The higher the shear rate, the shorter the response time. The data were measured for the same electric current (2 A), but the magnetization of the fluid sample was different. The response time $T_{90}$ ranges from 5.5 to 1.9 ms for shear rate $\dot{\gamma }$ from 11 to 218 s^−1^ (MRHCCS4-A and MRHCCS4-B). Within the measurement and evaluation error, it can be stated that both fluids are identical in terms of transient response. The effect of particle concentration is therefore nonsignificant. MRF 132-DG and MRC-C1L fluids exhibit a shorter response time $T_{90}$ than LR fluids in the range from 1.4 to 0.8 ms. This is probably due to the lower viscosity of the carrier liquid, which is about 3 times lower. The Fig. 5D shows the response time $T_{63}$ dependent on shear rate $\dot{\gamma }$. There can be seen the same trend as in the case of 90%. Koyanagi et al.^26^ experimentally determined the response time $\tau_{90}$ for ER fluid as 0.95 ms (dead time + time constant) which is near to our results. Lee et al.^32^ measured response time $\tau_{63}$ as 5.1 ms and $\tau_{90}$ as 6.1 ms for ER fluid in shear mode (data estimated from the publication graph). These values are slightly higher than the presented data in this paper. Laun and Gabriel^24^ measured the MR fluid response time based on sinusoidal excitation. The experiment determined the response time $\tau_{63}$ of MR fluid to 2.8 ms ± 0.5 ms at a shear rate of 100 s^−1^ at a magnetic flux density of 0.9 T. This measured value is about 3 times higher than the presented response time for Liquids Research fluids. However, it can be stated that the direct comparison of results is complicated because the measuring systems are not comparable. The studies^24,26^ used plate-plate configuration or study^32^ used rotating cylinder.

### Effect of magnetization and carrier fluid viscosity on the rheological response time

**Figure 6:**
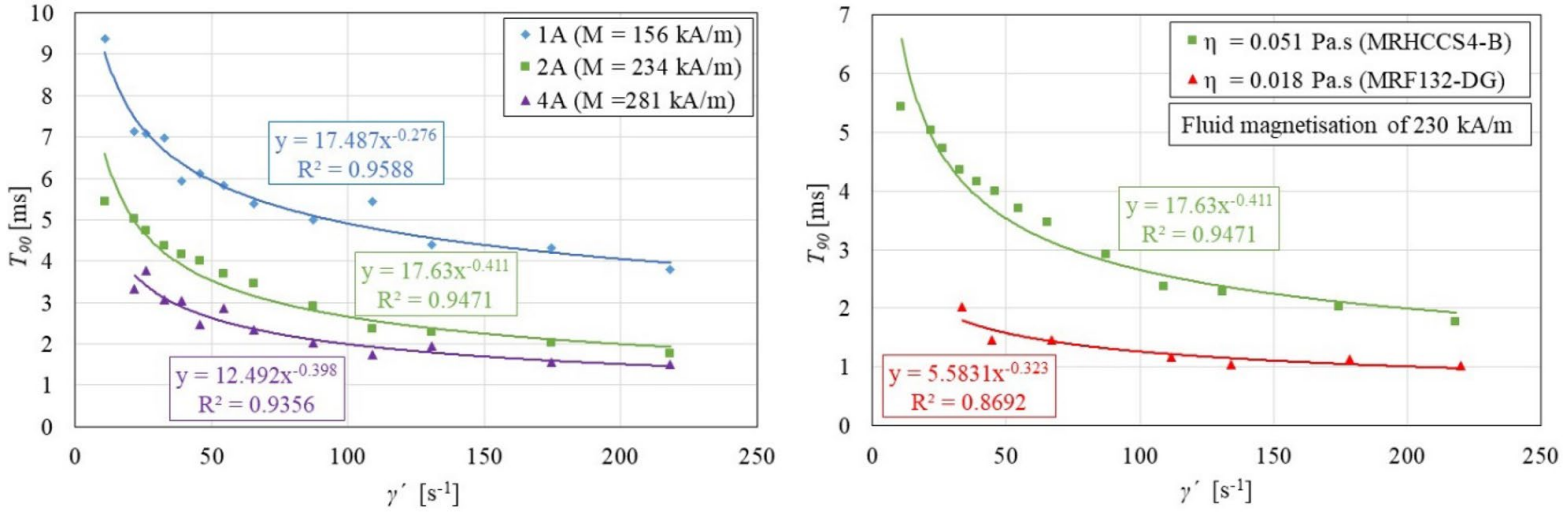
The effect of magnetization M (left) and carrier fluid viscosity η (right) on response time $T_{90}$.

The effect of fluid magnetization *M* on the response time *T*_*90*_ was demonstrated on MRHCCS4-B fluid because the effect of magnetization *M* was most noticeable. The fluid was measured at three levels of magnetization *M*, see Fig. 6, left. For all three levels of magnetization *M*, the dependence on the shear rate $\dot{\gamma }$ is exponential. It can be stated that the higher the magnetization *M* of the MR fluid, the lower the response time $T_{90}$. This is consistent with the theory.

The effect of carrier fluid viscosity *η* on response time $T_{90}$ will be demonstrated on two selected MR fluids (MRF 132-DG and MRHCCS4-B). These fluids have similar particle concentrations and different carrier fluid viscosities *η*. The viscosity of the MRHCCS4-B carrier fluid is approximately 2.8 times higher than the carrier fluid of MRF 132-DG. The right of Fig. 6 shows that an MR fluid with a higher carrier fluid viscosity *η* shows a significantly higher response time $\tau_{90}$. It should be noted that the MR fluids had the same magnetizations *M* of 230 kA/m, but different electric current excitation (MRF 132-DG electric current of 1.5 A; MRHCCS4-B electric current of 2 A). The effect of additives of carrier fluid viscosity was not considered here.

### Generalization of measured data

Sherman^14^ stated that MR fluid response time data in shear mode can be generalized using non-dimensional response time $T^{*}$ and Mason number $M_{n}$. This study provided the equation for the calculation of non-dimensional response time as:

3 $$T^{*} = \frac{{T_{90} }}{{\frac{144\eta }{{M^{2} \mu_{0} }}}}$$

**Figure 7:**
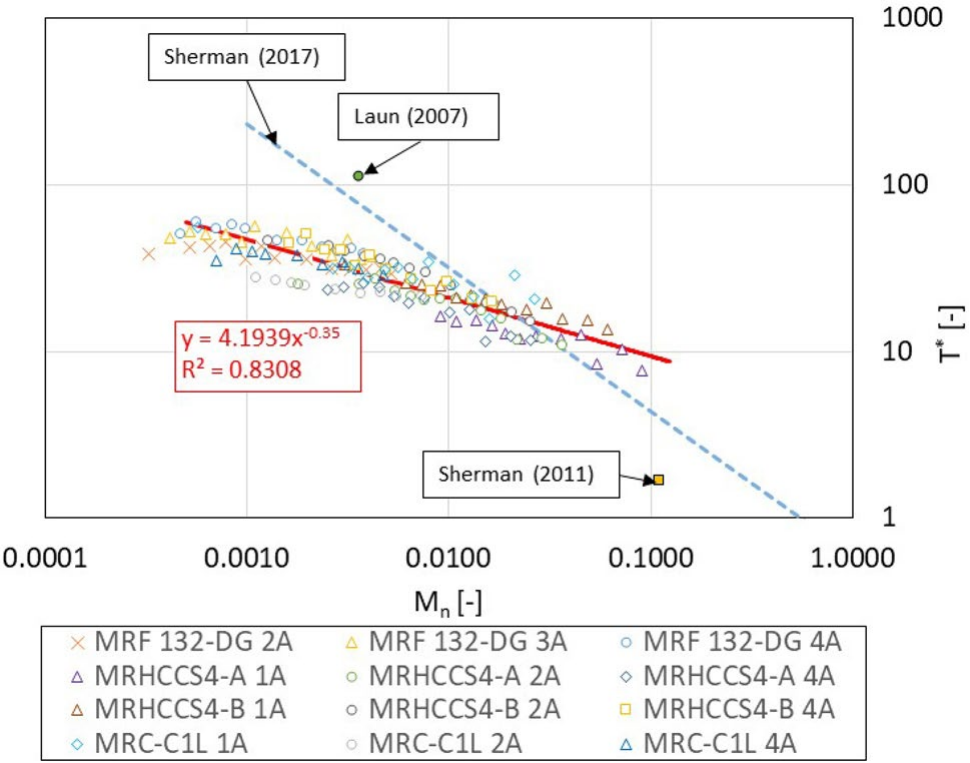
Dependency non-dimensional response time $T^{*}$ on Mason number $M_{n}$; Data from other publications have been estimated according to available information.

$T_{90}$ is the rheological response time (90%), $\eta$ is the viscosity of carrier fluid, $M$ is MR fluid magnetization and $\mu_{0}$ is vacuum permeability. The Mason number can be calculated as follow:

4 $$M_{n} = \frac{{144\eta \dot{\gamma }}}{{M^{2} \mu_{0} }}$$

where $\dot{\gamma }$ is shear rate. The Non-dimensional response time $T^{*}$ and Mason number $M_{n}$ were calculated from measured data, see Fig. 7. The master curve can be determined from measured data, see Fig. 7—red line. The results show a significant difference between the published model^14^ and our experiment for M_n_ values higher than 0.005. The $T^{*}$ and M_n_ was also evaluated (estimated) from papers^24,26^. This data is out of range of our measurement. However, it should be noted that the data obtained from the experiment are only from study^24^. The difference in the results may be due to (1) the model simplification and (2) inaccuracies in the measurement and evaluation of the measured data. It has been hypothesized that the difference may be due to the deformation of the measuring device (rheometer), which is not included in the model. This would result in a significant increase in response time $T_{90}$ at low shear rates $\dot{\gamma }$ compared to the model.

**Figure 8:**
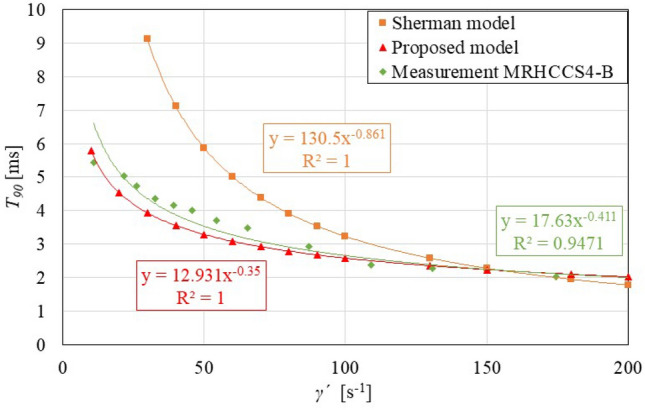
The comparison of model and experiment for the same inputs (MRHCCS4-B, electric current 2 A).

Figure 8 shows a comparison of the response time $T_{90}$ course on shear rate $\dot{\gamma }$ from the Sherman model, proposed model (Fig. 7 red) and from the experiment for MRHCCS4-B. The carrier fluid viscosity $\eta$, magnetization *M*, shear rates $\dot{\gamma }$ are the same for experiments and also for the model. It can be seen that the response time $T_{90}$ from experiments is significantly lower than that from the model. Thus, it can be stated that the possible deformation of the measuring device is not the source of the difference between the experiment and the model. The difference can be explained by certain simplifications of the model. However, both curves have an exponential character and therefore the model describes trends very well. Another significant difference is that measured MR fluid contains additives that are not included in the model. The question is how significant a difference can create this simplification. The surface roughness can also affect MR fluid dynamics^33^. This is also not included in the model, and can also play an important role.

## Conclusion

This paper deals with the experimental determination of magnetorheological fluid transient response (rheological response time) on the rapid change of a magnetic field in shear load mode. A unique rheometer was presented that allows almost unit step of magnetic fields and also allows the measuring of the development of MR fluid shear stress over time. The transient response was determined on four MR fluids that differ in supplier, particle concentration, or carrier fluid viscosity. The paper also includes a magnetic model and its experimental verification. The most important conclusions of the paper are the following:

- The response time of the magnetic field is $T_{90I} = 0.335$ ms and slightly increases with an increasing maximum value of electric current.
- The rise of shear stress exhibits an initial dead time of 0.4 ms, which is independent of the shear rate level.
- The value of the shear rate significantly influences the rheological response time at low shear rates. The higher the shear rate, the shorter the response time. The measured data of the response time can be fitted by a power-law function. The response time $T_{90}$ ranges from 5.5 to 1.9 ms for shear rate $\dot{\gamma }$ from 11 to 218 s^−1^ for MR fluid MRHCCS4-A and MRHCCS4-B.
- The fluid magnetization *M* significantly affects the rheological response time. The higher the magnetization *M* of the MR fluid, the lower the response time $T_{90}$.
- The carrier fluid viscosity also affects the rheological response time. The MR fluid with a higher carrier fluid viscosity $\eta$ shows a significantly higher response time $T_{90}$.
- All measured data was generalized in the term of non-dimensional response time $T^{*}$ and Mason number $M_{n}$. One master curve (T* = 4.1939M_n_^−0.35^) can be determined from measured data independent of magnetization *M*, carrier fluid viscosity $\eta$, shear rates $\dot{\gamma }$, etc. This is an important conclusion because the master curve allows the determination of rheological time response for a given MR fluid and given load (shear rates).

It should be noted that the our experimentally determined master curve shows a deviation from the model^14^. MR fluids used in the experiment and model differ in the type or concentration of additives (the model does not include additives), which may also affect the transient response. For this reason, a plan for further research in this area is to determine the rheological response time for homemade MR fluid (full control of additives) and measurement for a higher range of Mason numbers. We also see the potential for future research in the area of a particle chaining model that allows the showing of particle motion during the step change of a magnetic field.

## Data Availability

The data presented in this study are available on request from the corresponding author.
